# Two new species of *Symphylella* (Symphyla, Scolopendrellidae) from North China, with an analysis of genetic divergence in Chinese species

**DOI:** 10.3897/zookeys.1284.201089

**Published:** 2026-07-09

**Authors:** Ya-Li Jin, Yun Bu

**Affiliations:** 1 Natural History Research Center, Shanghai Natural History Museum, Shanghai Science & Technology Museum, Shanghai, 200041, China Natural History Research Center, Shanghai Natural History Museum, Shanghai Science & Technology Museum Shanghai China

**Keywords:** DNA barcoding, genetic distance, Myriapoda, Palaearctic, taxonomy

## Abstract

Symphylans from Liaoning Province, the Inner Mongolia Autonomous Region, and Shanxi Province, North China, were investigated and studied for the first time. Two new species of the *Symphylella isabellae* group, *S. hylophila***sp. nov**. and *S. rotundata***sp. nov**., are identified and described. *Symphylella hylophila***sp. nov**. is characterized by having blunt ends of the processes, long, subconical styli each with a blunt apex, and the absence of long, erect setae on the cerci. *Symphylella rotundata***sp. nov**. is characterized by having noticeably roundish ends of the tergal processes, short, subconical styli each with a pointed apex, and long, erect setae on the outer and ventral sides of the cerci. These new species are carefully delimited from similar species. Morphological analyses were supported by DNA barcodes, which were used to analyse the genetic divergence of Chinese species of the genus *Symphylella*.

## Introduction

Symphylans are a group of common soil arthropods (class Symphyla) with about 250 species known worldwide further divided into two families (Szucsich and Scheller 2011; Jin et al. 2023). *Symphylella* Silvestri, 1902 is a cosmopolitan genus of the family Scolopendrellidae Bagnall, 1913 with 68 known species (Jin and Bu 2025). Knowledge on *Symphylella* in China remains limited, with only 10 species reported until now (Jin and Bu 2023, 2025). Integrative taxonomy, combining evidence from different lines of evidence in taxonomic decisions (Pante et al. 2015), is hampered in Symphyla by the lack of DNA-based information for the greater part of described species. First attempts to provide standard DNA barcodes by sequencing the Folmer region of the mitochondrial COI gene were, however, performed only recently (Jin and Bu 2023, 2025).

## Materials and methods

**Figure 1.**
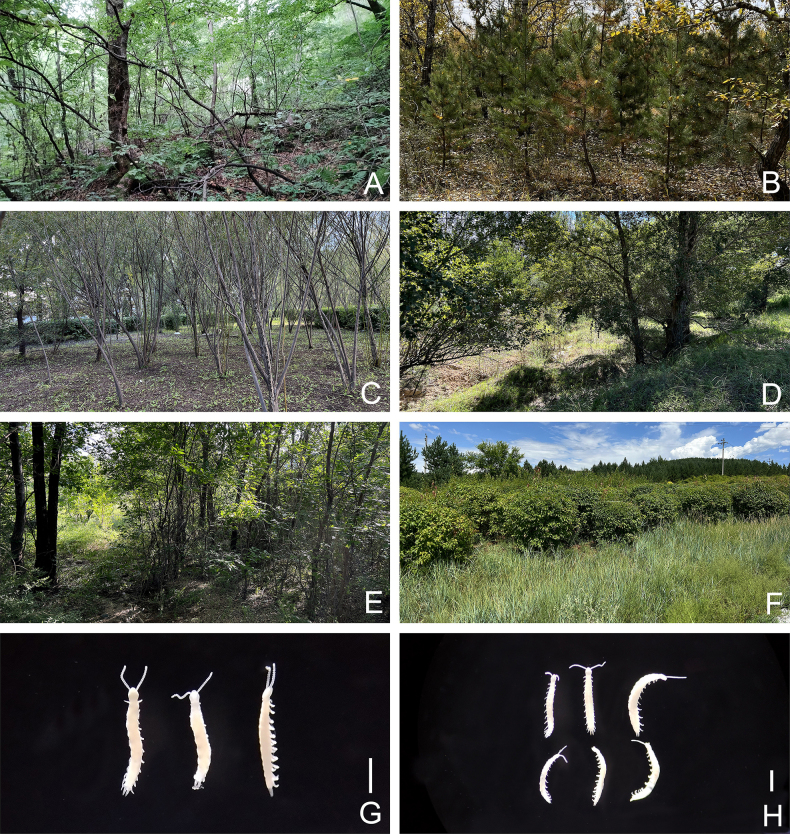
Habitats of collection sites and specimens in alcohol of two new species. **A**. Habitat of *Symphylella hylophila* sp. nov.: broad-leaf forest of the Heshangmao Nature Reserve, Benxi City, Liaoning Province; **B–F**. Habitats of *Symphylella rotundata* sp. nov.: **B**. Mixed forest in Datong City, Shanxi Province; **C**. Artificial bush forest in Tongliao City, Inner Mongolia Autonomous Region; **D**. Broad-leaf forest in Tongliao City, Inner Mongolia Autonomous Region; **E**. Broad-leaf forest in Hohhot City, Inner Mongolia Autonomous Region; **F**. Bush forest in Xilinhot City, Inner Mongolia Autonomous Region; **G**. Specimens of *Symphylella hylophila* sp. nov. in alcohol; **H**. Specimens of *Symphylella rotundata* sp. nov. in alcohol. Scale bars: 1 mm.

During a soil-fauna investigation in Liaoning Province, the Inner Mongolia Autonomous Region, and Shanxi Province, plenty of symphylans were collected. Specimens were extracted from soil and litter samples of forests by using Berlese-Tullgren funnels and subsequently preserved in 80% ethanol. Their habitats are shown in Fig. 1A–F. Specimens were mounted on slides in Hoyer’s solution and dried in an oven at 50 °C. Observations were carried out under a phase contrast microscope (Leica DM 2500). Photographs were taken with a digital camera mounted on a microscope (Leica DMC 4500). Line drawings were made with the aid of a drawing tube and vectorized with SAI v. 2.0 software. All specimens are deposited in the collections of the Shanghai Natural History Museum (**SNHM**), Shanghai, China.

Abbreviations used in the descriptions: **a0** – antero-central seta on the head, **als** – anterolateral setae on tergites, **as** – apical seta on the process, **bo** – bladder-shaped organs on antenna, **co** – cavity-shaped organs on antenna, **cs** – central setae on tergites, **ibs** – inner basal setae on the process, **is** – inserted setae on the process, **lm** – lacinia mobilis on the mandible, **lms** – lateromarginal setae on tergites, **pi** – pars incisivus on the mandible, **pm** – pars molaris on the mandible, **rso** – rudimentary spined sensory organs on antenna, **so** – spined sensory organs on antenna.

Specimens used for DNA extraction were preserved in absolute ethanol at −20 °C. Prior to DNA extraction, each specimen was identified under a stereomicroscope. For DNA barcoding, total genomic DNA was extracted from the whole body of a single individual using the Promega genomic DNA purification kit following the manufacturer’s instructions. The primer pair LCO (5’–GGTCAACAAATCATAAAGATATTGG–3’) and HCO (5’–TAAACTTCAGGGTGACCAAAAAATCA–3’) (Folmer et al. 1994) was used for amplification and sequencing.

To analyse the genetic divergences between the new species and their congeners, DNA barcodes from eight specimens of the two new species were sequenced. The corresponding sequences of eight species of *Symphylella* and the outgroup, *Scutigerella sinensis* Jin & Bu, 2023, were downloaded from GenBank for joint analysis. The detailed information and accession numbers of all sequences analysed in this study are listed in Table 1. A neighbour-joining tree based on COI sequences was built by using MEGA X (Kumar et al. 2018) corrected with the K2P model (Kimura 1980). The accuracy of the results was tested by 1,000 bootstrap replicates. The genetic distance (K2P-distance) was calculated using MEGA X (Kimura 1980; Kumar et al. 2018).

**Table 1.** Taxonomic and collection information of the species used in the analysis.

**Species**	**Voucher**	**Locality**	**GenBank number**	**Reference**
*Symphylella hylophila***sp. nov**.	LN-BX-2023014	China: Liaoning	PZ433329	Present study
*Symphylella hylophila***sp. nov**.	LN-BX-2023015	China: Liaoning	PZ433330	Present study
*Symphylella rotundata***sp. nov**.	NM-2025018	China: Inner Mongolia	PZ433331	Present study
*Symphylella rotundata***sp. nov**.	NM-2025019	China: Inner Mongolia	PZ433332	Present study
*Symphylella rotundata***sp. nov**.	NM-2025020	China: Inner Mongolia	PZ433333	Present study
*Symphylella rotundata***sp. nov**.	NM-2025021	China: Inner Mongolia	PZ433334	Present study
*Symphylella rotundata***sp. nov**.	NM-2025022	China: Inner Mongolia	PZ433335	Present study
*Symphylella rotundata***sp. nov**.	SX-DT-2023004	China: Shanxi	PZ433336	Present study
*Symphylella communa*	JS-WX-2021010	China: Jiangsu	PX169704	Jin and Bu 2025
*Symphylella flabella*	CQ-YTL-2022007	China: Chongqing	PX169697	Jin and Bu 2025
*Symphylella macrochaeta*	SH-JZGY-2021009	China: Shanghai	PX169702	Jin and Bu 2025
*Symphylella macrochaeta*	ZJ-ZS-2020011	China: Zhejiang	PX169703	Jin and Bu 2025
*Symphylella micropora*	CQ-JYS-2022001	China: Chongqing	PX169698	Jin and Bu 2025
*Symphylella micropora*	CQ-YTL-2022009	China: Chongqing	PX169700	Jin and Bu 2025
*Symphylella minuta*	JS-WX-2021008	China: Jiangsu	PX169705	Jin and Bu 2025
*Symphylella obtusa*	CQ-JYS-2021002	China: Chongqing	PX169693	Jin and Bu 2025
*Symphylella obtusa*	CQ-JYS-2021012	China: Chongqing	PX169694	Jin and Bu 2025
*Symphylella yintiaolingensis*	CQ-YTL-2022004	China: Chongqing	PX169695	Jin and Bu 2025
*Symphylella* sp.	YG-2006	China: Jiangsu	NC011572	Gai et al. 2008
*Scutigerella sinensis*	JYL-DJS2017011	China: Shanghai	OQ165321	Jin and Bu 2023

## Results

Among the Symphyla found in Liaoning Province, the Inner Mongolia Autonomous Region, and Shanxi Province, representatives of *Symphylella* could not be assigned to any known described species. Thus, two new species are described and compared to closely related species. Standard DNA barcodes of the new species were sequenced to analyse the genetic divergence among Chinese representatives of the genus *Symphylella*. These data provide the first insights into the genetic divergence among *Symphylella* in China.

### Taxonomy

#### Family Scolopendrellidae Bagnall, 1913

##### Genus Symphylella

Taxon classificationAnimaliaSymphylaScolopendrellidae

Silvestri, 1902

454DE608-EE2F-5979-B845-A6E164B065B0

###### Type species.

*Symphylella isabellae* (Grassi, 1886); type locality: southern Italy.

##### Symphylella hylophila sp. nov.

Taxon classificationAnimaliaSymphylaScolopendrellidae

B5002C11-0AD7-5685-B09C-AD3C40607464

https://zoobank.org/905B6422-4080-42A6-80AC-C0215FA098FA

**Figure 2.**
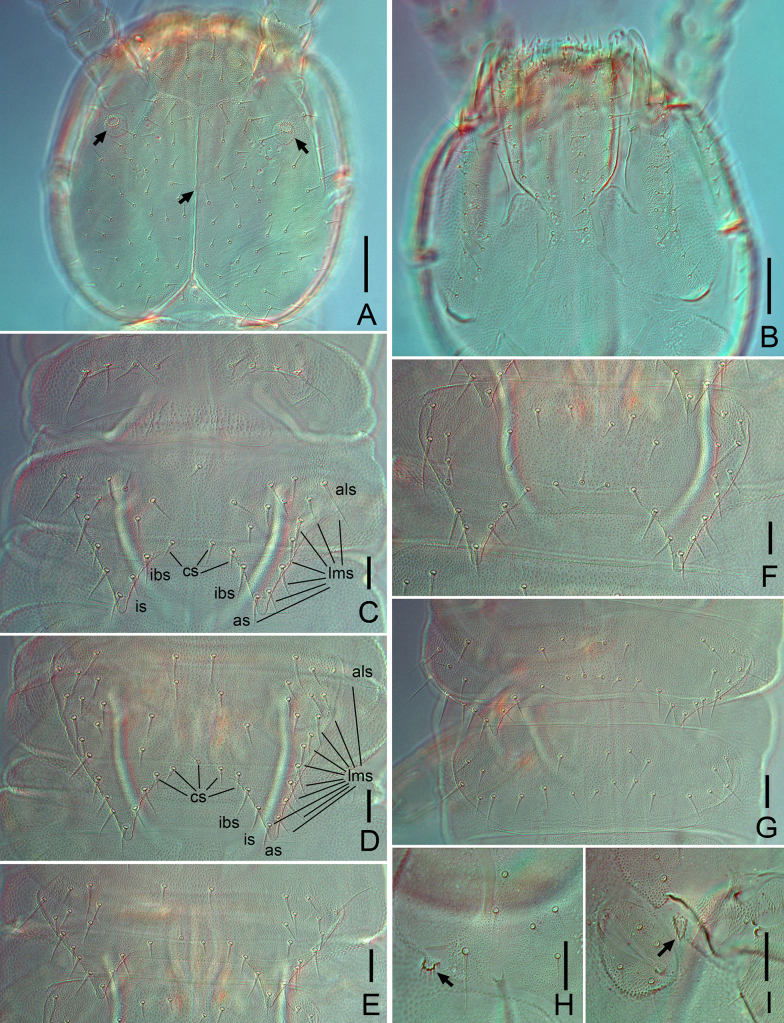
*Symphylella hylophila* sp. nov. **A**. Head, dorsal view (arrows indicate Tömösváry organ and node-like interruption); **B**. Head, ventral view; **C**. Tergites 1–2 (*als*–anterolateral seta, *as*–apical seta, *cs*–central seta, *ibs*–inner basal seta, *is*–inserted seta, *lms*–lateromarginal setae); **D**. Tergite 3; **E**. Tergite 4; **F**. Tergite 5; **G**. Tergites 13–14; **H**. Leg 1, left side (arrow indicates reduced leg); **I**. Right stylus and coxal sac at the base of leg 8 (arrow indicates stylus). Scale bars: 50 μm (**A, B**); 20 μm (**C–I**).

**Figure 3.**
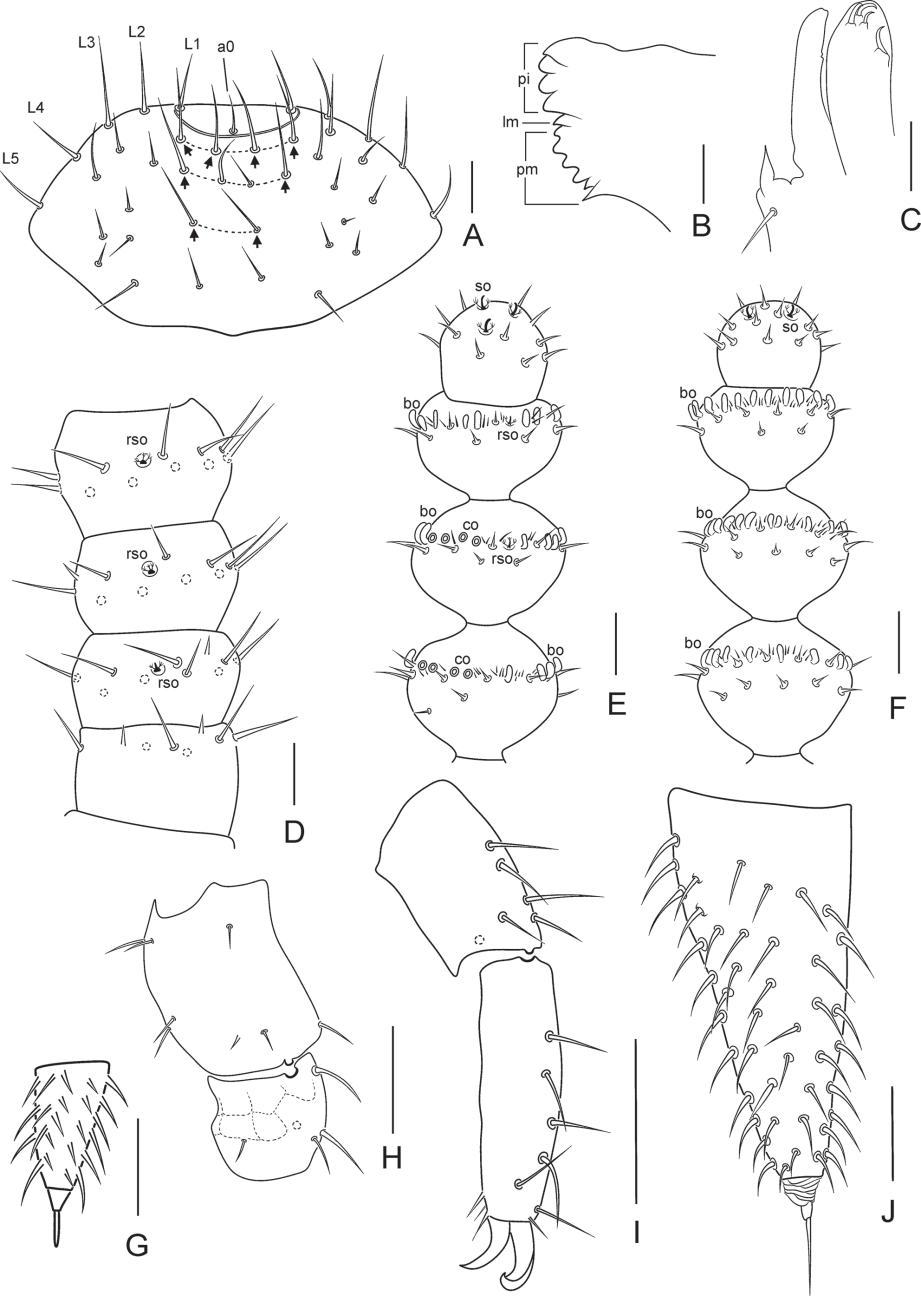
*Symphylella hylophila* sp. nov. **A**. Frons (L1–L5–lateral setae, a0–antero-central seta, arrows indicate macrosetae); **B**. Right mandible, lateral view (*pi*–pars incisivus, *pm*–pars molaris, *lm*–lacinia mobilis); **C**. Left first maxilla; **D**. Left 1–4 antennomeres, dorsal view (*rso*–rudimentary spined sensory organ); **E**. Right terminal four antennomeres, dorsal view (*bo*–bladder-shaped organ, *co*–cavity-shaped organ, *so*–spined sensory organ); **F**. Right terminal four antennomeres, ventral view; **G**. Left stylus at base of leg 6; **H**. Trochanter and femur of leg 12, right side, ventral view; **I**. Tibia and tarsus of leg 12, right side, dorsal view; **J**. Left cercus, dorsolateral view. Scale bars: 20 μm (**A–F**, **H–J**); 5 μm (**G**).

Figs 1G, 2, 3, 3 Tables 2–4

###### Diagnosis.

*Symphylella hylophila* sp. nov. belongs to the isabellae group in which inserted setae are present on the process of the tergite (Jin and Bu 2025). The new species is characterized by having blunt ends of the processes, long, subconical styli with a blunt apex, and the absence of long, erect setae on the cerci.

###### Type material.

***Holotype***: • male (SNHM slide no. LN-BX-SY2023003), China, Liaoning Province, Benxi City, Heshangmao Nature Reserve, extracted from soil samples of broad-leaf deciduous forest (Fig. 1A), alt. 510 m, 41°06'N, 124°13'E, 26-VII-2023, coll. Y. Bu, Y.L. Jin & S.Q. Yang. ***Paratypes***: (2 females, 8 males) • 2 females (SNHM slides no. LN-BX-SY2023002, LN-BX-SY2023006), same data as holotype • 4 males (SNHM slides no. LN-BX-SY2023001, LN-BX-SY2023004, LN-BX-SY2023005, LN-BX-SY2023007), same data as holotype • 4 males (SNHM slides no. LN-BX-SY2023009–LN-BX-SY2023012), ibidem, 27-VII-2023, coll. Y. Bu, Y.L. Jin & S.Q. Yang.

###### Description.

Adult body 2.8 mm long on average (2.4–3.5 mm, *n* = 11) (Fig. 1G), holotype 2.8 mm.

***Head*** length 263–300 μm, width 255–300 μm, with widest part at the same level as points of articulation of mandibles. Central rod (145–160 μm) distinct and divided into two parts by a node-like interruption at half length. Dorsal side of head moderately covered with setae of different lengths (7–22 μm) (Fig. 2A). Frons with 5+5 lateral setae (20–30 μm), eight macrosetae (24–33 μm) arranged as 4/2/2 (counted from anterior row to posterior row) and 1.3–2.3 times as long as *a0* (12–18 μm) (Fig. 3A), and 18–20 normal setae. Head cuticle with fine granulation on dorsal side and coarse granulation on lateral and ventral sides (Fig. 2A, B).

***Tömösváry organ*** globular, diameter 19–21 μm, shorter than half of greatest diameter of third antennomere (43–50 μm); aperture round (9–12 μm), with distinct vertical inner striae (Fig. 2A).

***Mouthparts***. Mandible composed of three parts: *pi* with four distinct thick teeth, *pm* with four blunt teeth and two proximal acute teeth, and *lm* with one subconical process observed in lateral view (Fig. 3B). First maxilla with two lobes; inner lobe with four hooked teeth; palp pointed and sharp (Fig. 3C). Anterior part of second maxilla with many small protuberances, each carrying one seta; distal setae thick; posterior part of second maxilla with sparse setae (Fig. 2B). Cuticle of second maxilla covered with dense pubescence.

***Antennae*** with 14–27 antennomeres (18 in holotype), about 0.2 of body length. First antennomere cylindrical, 1.3–1.7 times as wide as long (length 25–34 μm, width 34–48 μm), with 5–7 setae in one whorl; longest inner seta 21–23 μm, 1–3 minute setae on antennomere (Fig. 3D). Second antennomere wider (41–48 μm) than long (24–31 μm), with 7–10 setae inserted around antennal wall; interior setae (19–23 μm) longer than exterior ones (16–17 μm) (Fig. 3D). Chaetotaxy of third antennomere similar to that of preceding ones (Fig. 3D). Setae on proximal antennomeres longer than those on distal antennomeres. Proximal antennomeres with only primary whorl of setae, on middle and subapical antennomeres with several minute setae in secondary whorl (Fig. 3E, F). Four kinds of sensory organs observed on antenna (Fig. 3D–F): *rso* on dorsal side from second to subterminal antennomeres; *so* only present on apical antennomere; *co* on dorsal side of antennomeres 5–8 to penultimate or antepenultimate antennomere, increasing in number to a maximum of five on subterminal antennomere; *bo* irregular, oblate, or slightly curved, present on antennomeres 8–12 to penultimate, increasing in number to a maximum of 23 on penultimate antennomere. Apical antennomere subspherical, somewhat wider than long (width 30–37 μm, length 23–32 μm), with five *so* and 18–22 short setae apically (Fig. 3E, F). All antennomeres covered with short pubescence. Chaetotaxy and sensory organs of antennae of holotype are given in Table 2.

**Table 2.** Numbers of setae and sensory organs on antennae of *Symphylella hylophila* sp. nov. (holotype, excluding apical antennomere).

**Antennomere**	**Primary whorl setae**	**Secondary whorl setae**	**Rudimentary spined sensory organs**	**Cavity-shaped organs on dorsal side**	**Bladder-shaped organs**
1	7				
2	9		1		
3	10		1		
4	11		1		
5	11		1	1	
6	11		1	1	
7	13	3		1	
8	12	4		1	
9	12	6		1	3
10	11	8		1	4
11	12	7	1	1	5
12	13	9	1	2	6
13	13	7	1	2	8
14	13	7		2	12
15	11	9		4	14
16	10	7	1	4	19
17	10	8	1		21

***Trunk***. Length from base to tip of triangular processes almost same as or slightly longer than its basal width (Fig. 2C, D, F), except for tergites 4, 7, 10, and 13 (shorter than basal width) (Fig. 2E, G). Basal distance between processes of tergites distinctly longer than process length, except for tergites 2 (same as or a little shorter than process length) (Table 3). All processes with blunt, semicircular ends. Definition of chaetotaxy on tergite as follows: *als* located on anterolateral angle of each tergite; *as* close to process apex; *lms* located on lateral margin of process and including *als* and *as*; *ibs* located on inner base of processes; *is* present between *ibs* and *as*; *cs* present at base of processes between *ibs*; other setae including all setae except those above nominated ones (Fig. 2C, D). Anterolateral setae (32–50 μm) on tergites 2, 3, 4, 6, 7, 9, 10, and 12 distinctly longer than other *lms* of same tergite (Fig. 2C), while those on tergites 5, 8, and 11 (20–31 μm) shorter than longest second *lms* of same tergite (Fig. 2F). Most processes with 0–2 *is* (seldom 3). All tergites pubescent (Fig. 2C–G).

**Table 3.** Measurements of tergites and processes of *Symphylella hylophila* sp. nov. (holotype in brackets, in μm).

**Tergite**	**Length**	**Width**	**Length of processes**	**Basal width of processes**	**Basal distance between processes**
1	25–40 (30)	140–175 (140)			
2	55–80 (70)	170–205 (170)	35–50 (38)	35–50 (37)	35–57 (38)
3	100–157 (113)	180–250 (198)	38–47 (43)	38–50 (40)	48–70 (48)
4	58–82 (70)	228–290 (230)	30–43 (35)	35–50 (45)	63–103 (63)
5	65–80 (73)	213–250 (213)	38–43 (40)	38–43 (38)	83–107 (83)
6	125–150 (138)	255–325 (255)	40–52 (45)	40–52 (43)	75–102 (75)
7	72–88 (83)	258–350 (258)	33–37 (35)	40–50 (43)	87–147 (87)
8	73–100 (88)	238–280 (238)	38–50 (43)	38–50 (40)	92–112 (92)
9	103–163 (138)	280–350 (280)	40–62 (40)	38–62 (38)	88–100 (88)
10	75–103 (80)	275–350 (275)	30–42 (30)	38–60 (38)	100–165 (100)
11	63–98 (80)	243–300 (243)	33–50 (33)	38–50 (38)	100–132 (100)
12	118–147 (125)	270–347 (282)	41–45 (41)	43–52 (43)	98–112 (101)
13	67–85 (70)	255–313 (255)	18–30 (18)	33–62 (33)	100–125 (103)
14	62–70 (63)	228–272 (228)			
15	87–170 (87)	240–312 (240)	28–30 (30)	38–55 (43)	67–112 (68)
16	55–88 (55)	205–237 (205)			
17	113–162 (113)	183–212 (187)			

***Tergites***. Tergite 1 reduced, with 4+4 setae (Fig. 2C), asymmetrically lacking one seta in one paratype. Tergite 2 complete, with two triangular posterior processes, 6–8 *lms*, one or two *is*, two or three *cs*, *als* 0.8–1.2 of length of process, processes 0.9–1.0 time as long as broad, basal distance between processes 0.8–1.5 times as long as process length (Fig. 2C). Tergite 3 complete, broader and longer than preceding one, with ratios of 0.8–1.1, 0.9–1.1, and 1.1–1.5, respectively, 8–10 *lms*, 1–2 *is*, 2–5 *cs* (Fig. 2D). Tergite 4 broader than tergite 3, with ratios 1.1–1.5, 0.8–1.1, and 1.8–3.0, respectively, 6–7 *lms* (5 *lms* in one paratype), 0–1 *is*, 3–5 *cs* (Fig. 2E). Chaetotaxy of tergites 5–7, 8–10, and 11–13 similar to tergites 2–4 (Fig. 2F, G). Pattern of alternating tergite lengths of two short tergites followed by one long tergite only disrupted at caudal end. Tergites 14, 16, and 17 without processes, with 18–29, 12–19, and 19–42 setae, respectively. Measurements and chaetotaxy of tergites are given in Tables 3, 4, respectively.

***Legs***. First pair of legs reduced to two small hairy cupules, each with one long seta (11–12 μm) (Fig. 2H). Basal areas of legs 2–12 each with 3–9 setae. Leg 12 slightly shorter than head length, trochanter 1.2–1.5 times longer than wide (58–68 μm, 43–53 μm), with seven or eight subequal setae in total, longest seta 12–17 μm (Fig. 3H); femur almost as long as wide (38–43 μm, 38–40 μm), with five setae, longest outer seta (17–22 μm) 0.5–0.6 of greatest diameter of podomere in length, dorsally with cuticular thickenings in pattern of scales (Fig. 3H); tibia 1.2–1.8 times longer than wide (43–50 μm, 28–43 μm), with six or seven setae, longest outer one (18–23 μm) 0.5–0.7 of greatest diameter of tibia (Fig. 3I); tarsus sub-cylindrical, 2.6–3.9 times as long as wide (55–73 μm, 18–21 μm) with six dorsal setae: four straight and protruding, two depressed, longest seta (15–19 μm) shorter than greatest diameter of tarsus, three or four minute setae close to claw (Fig. 3I). Claws slightly curved, anterior one broader and more curved than posterior one (Fig. 3I). All legs covered with dense pubescence except areas with cuticular thickenings.

***Coxal sacs*** present at bases of legs 3–9, fully developed, each with 3–5 setae on surface (Fig. 2I). Corresponding areas of legs 2, 10, 11, and 12 replaced by 1–3 setae, respectively.

***Styli*** present at base of legs 3–12, long and subconical (length 9–13 μm, width 4–5 μm), basal part with straight hairs; distal part hairless, with blunt apex (Figs 2I, 3G).

***Sense calicles*** with smooth margin around pit. Sensory seta inserted in cup centre, extremely long (120–160 μm).

***Cerci*** about 0.5–0.6 of head in length, 2.6–3.2 times as long as its greatest width (138–195 μm, 45–63 μm), moderately covered with 62–97 setae. All setae slightly curved and almost equal length, without long and erect setae. Longest seta (17–25 μm) shorter than half of greatest width of cerci, terminal area short (20–25 μm), circled by 8–11 layers of curved ridges. Terminal seta (18–25 μm) close in length with terminal area (Fig. 3J).

###### Etymology.

Derive from Greek words *hylē*, meaning forest, and *philos*, meaning “loving”. The species name, *hylophila*, is feminine and refers to its forest habitat.

###### Distribution.

Only known from the type locality in Liaoning, China.

###### Remarks.

*Symphylella hylophila* sp. nov. is very similar to the widespread *S. vulgaris* (Hansen, 1903) in sharing a similar shape and chaetotaxy of the processes and of leg 12, and it is characterized by the absence of erect lateral setae on the cercus. The two species can be distinguished by the chaetotaxy of tergite 1 (4+4 setae in *S. hylophila* sp. nov. vs 3+3 setae *S. vulgaris*), the tiny projection close to the posterior margin of the head (absent in *S. hylophila* sp. nov. vs present in *S. vulgaris*), and the position of apical setae on the processes (not very close to the apical end in *S. hylophila* sp. nov. vs very close to the apical end in *S. vulgaris*).

##### Symphylella rotundata sp. nov.

Taxon classificationAnimaliaSymphylaScolopendrellidae

C8149E8A-54B0-5BDB-8AEA-FE8832223209

https://zoobank.org/17ECE803-405B-4017-A83E-C83F15A1A4E2

**Figure 4.**
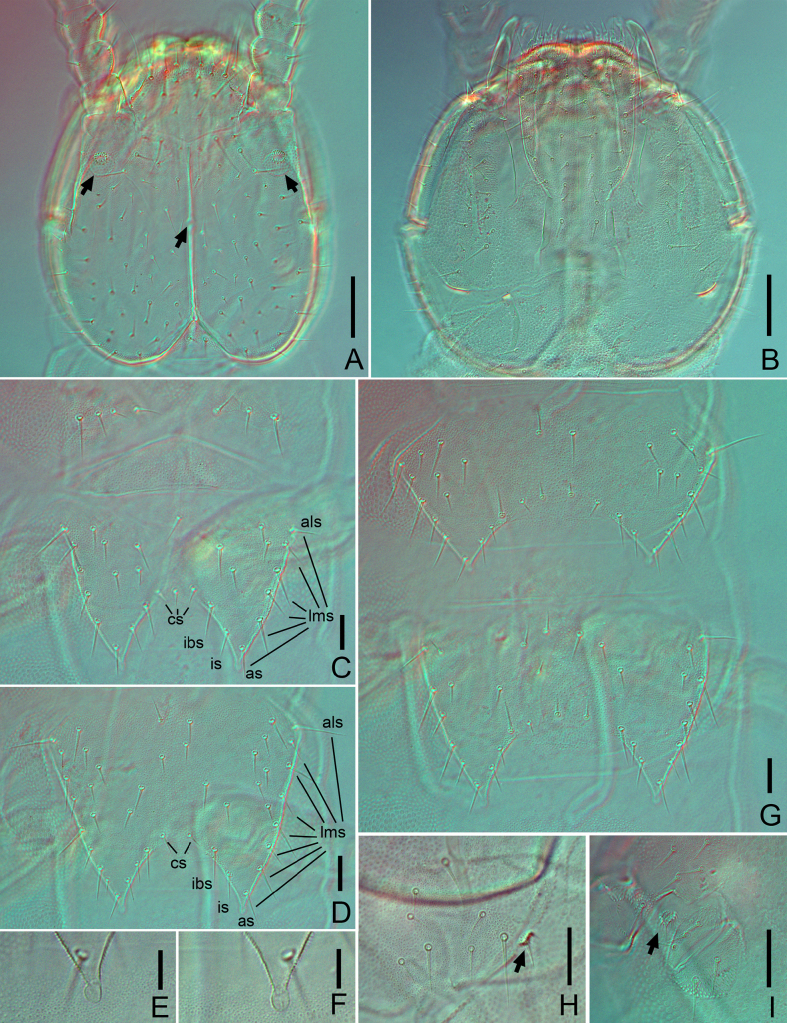
*Symphylella rotundata* sp. nov. **A**. Head, dorsal view (arrows indicate Tömösváry organ and node-like interruption); **B**. Head, ventral view; **C**. Tergites 1–2 (*als*–anterolateral seta, *as*–apical seta, *cs*–central seta, *ibs*–inner basal seta, *is*–inserted seta, *lms*–lateromarginal setae); **D**. Tergite 3; **E**. End of process on tergite 3, left side; **F**. End of process on tergite 3, right side; **G**. Tergites 4–5; **H**. Leg 1, right side (arrow indicates reduced leg); **I**. Left stylus and coxal sac at the base of leg 3 (arrow indicates stylus). Scale bars: 50 μm (**A, B**); 20 μm (**C, D, G–I**); 10 μm (**E, F**).

**Figure 5.**
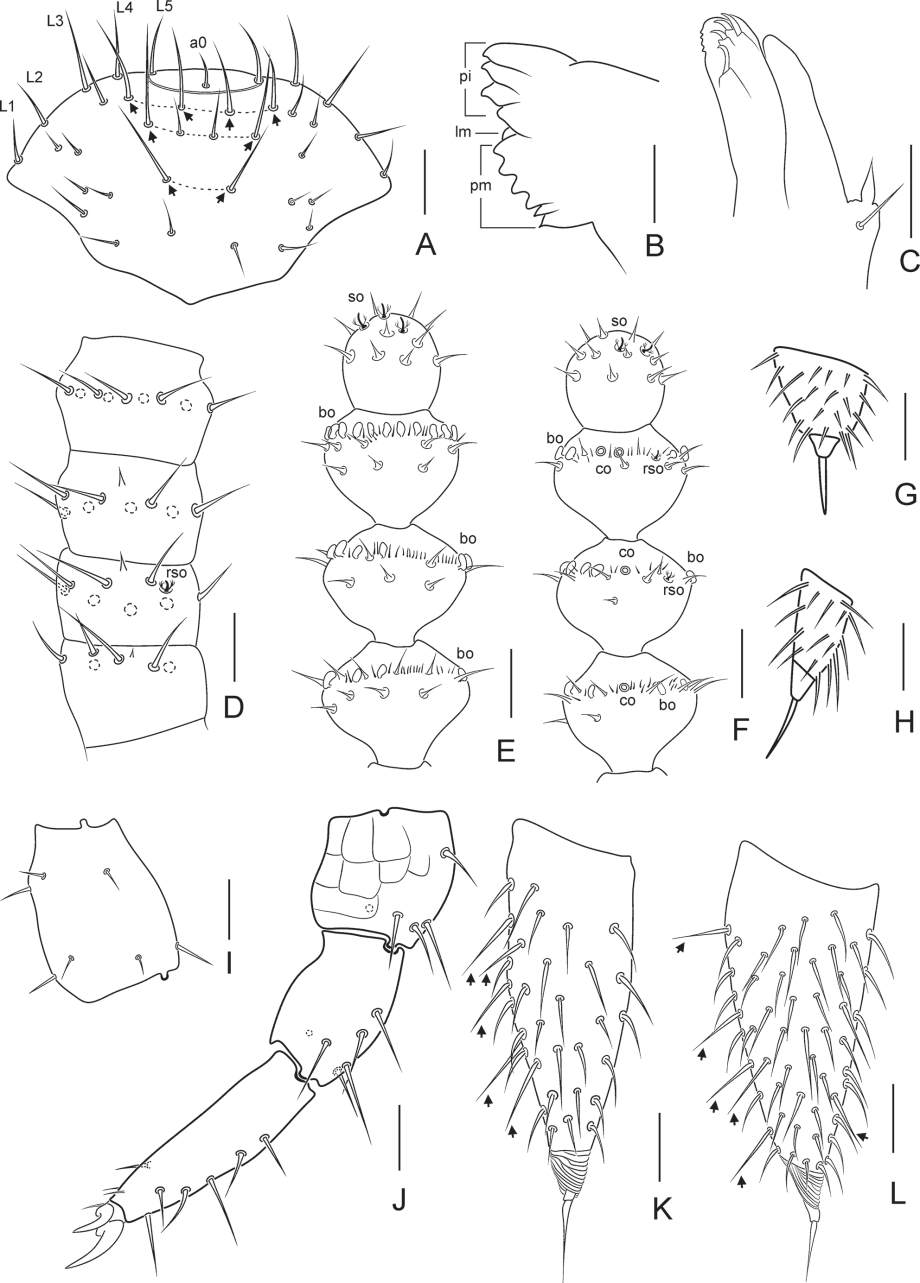
*Symphylella rotundata* sp. nov. **A**. Frons (L1–L5–lateral setae, a0–antero-central seta, arrows indicate macrosetae); **B**. Right mandible, lateral view (*pi*–pars incisivus, *pm*–pars molaris, *lm*–lacinia mobilis); **C**. Left first maxilla; **D**. Right 1–4 antennomeres, dorsal view (*rso*–rudimentary spined sensory organ); **E**. Right terminal four antennomeres, ventral view (*bo*–bladder-shaped organ, *so*–spined sensory organ); **F**. Right terminal four antennomeres, dorsal view (*co*–cavity-shaped organ); **G**. Right stylus at the base of leg 4; **H**. Left stylus at the base of leg 12; **I**. Trochanter of leg 12, right side, ventral view; **J**. Femur, tibia, and tarsus of leg 12, right side, dorsal view; **K**. Left cercus, dorsal view (arrows indicate long and erect setae); **L**. Left cercus, lateral view (arrows indicate long and erect setae). Scale bars: 20 μm (**A–F**, **I**–**L**); 5 μm (**G, H**).

Figs 4, 5, Tables 5–7

###### Diagnosis.

*Symphylella rotundata* sp. nov. belongs to the isabellae group (Jin and Bu 2025). It is characterized by having noticeably roundish ends of the tergal processes, a short, subconical stylus with a pointed apex, and long, erect setae on the outer and ventral sides of the cerci.

###### Material examined.

***Holotype***: • female (SNHM slide no. NM-SY2025005), China, Inner Mongolia Autonomous Region, Tongliao City, Naiman Banner, extracted from soil samples in an artificial bush forest of a park (Fig. 1C), alt. 337 m, 42°51'N, 120°39'E, 3-VIII-2025, coll. Y.L. Jin. ***Paratypes***: (19 females) • 3 females (SNHM slides no. SX-SY2023001–SX-SY2023003), China, Shanxi Province, Datong City, extracted from litter in mixed forest (Fig. 1B), 40°23'N, 113°13'E, 4-X-2023, coll. Y.L. Jin • 1 female (SNHM slide no. NM-SY2025001), China, Inner Mongolia Autonomous Region, Xilingol League, Sonid Right Banner, extracted from soil samples of a city park, alt. 1106 m, 42°43'N, 112°37'E, 23-VII-2025, coll. Y.L. Jin • 2 females (SNHM slides no. NM-SY2025002–NM-SY2025003), China, Inner Mongolia Autonomous Region, Xilingol League, Xilinhot City, Nanshan Forest Park, extracted from soil samples in bush forest (Fig. 1F), alt. 1007 m, 43°52'N, 116°4'E, 31-VII-2025, coll. Y.L. Jin • 2 females (SNHM slides no. NM-SY2025004, NM-SY2025006), same data as holotype • 3 females (SNHM slides no. NM-SY2025007–NM-SY2025009), China, Inner Mongolia Autonomous Region, Tongliao City, Kulun Banner, extracted from soil samples in broad-leaf forest (Fig. 1D), alt. 356 m, 42°44'N, 121°27'E, 4-VIII-2025, coll. Y.L. Jin • 1 female (SNHM slide no. NM-SY2025010), China, Inner Mongolia Autonomous Region, Tongliao City, Kulun Banner, Hada Botanical Garden, extracted from soil samples in broad-leaf forest, alt. 364 m, 42°45'N, 121°45'E, 4-VIII-2025, coll. Y.L. Jin • 7 females (SNHM slides no. NM-SY20250011–NM-SY20250017), China, Inner Mongolia Autonomous Region, Hohhot City, Daqing Mountain, extracted from soil samples in broad-leaf forest (Fig. 1E), alt. 1392 m, 40°55'N, 111°43'E, 6-VIII-2025, coll. Y.L. Jin.

###### Description.

Adult body 2.7 mm long on average (2.2–3.3 mm, *n* = 20) (Fig. 1H), holotype 3.2 mm.

***Head*** longer than wide, length 225–275 μm, width 195–228 μm, with widest part at same level as points of articulation of mandibles. Central rod well developed, divided into two portions by a node-like interruption, with anterior part 58–75 μm and posterior part 65–80 μm. Dorsal side of head moderately covered with setae of different length (8–27 μm) (Fig. 4A). Frons with 5+5 lateral setae (13–35 μm); macrosetae (20–45 μm) arranged as 4/2/2 and 1.3–2.5 times as long as *a0* (14–19 μm), and 17–19 normal setae (Fig. 5A).

***Tömösváry organ*** globular, diameter 15–20 μm, about 0.4–0.6 time of greatest diameter of third antennomere (31–40 μm), aperture subround, 4–10 μm, with inner wall striated (Fig. 4A).

***Mouthparts***. Mandible composed of three parts: *pi* with four distinct thick teeth, *pm* with four smaller teeth and two pointed teeth, and *lm* with one little process observed in lateral view (Fig. 5B). First maxilla with two lobes; inner lobe with four teeth; palp of first maxilla sharp (Fig. 5C). Anterior part of second maxilla with many small protuberances, each carrying one seta, distal setae thick; posterior part with sparse setae. Cuticle of second maxilla covered with dense pubescence (Fig. 4B).

***Antennae*** with 15–21 antennomeres (17 on left and 15 on right in holotype), about 0.2 of body length. First antennomere cylindrical, 1.0–1.5 time as wide as long (width 30–35 μm, length 23–30 μm), with 6 setae, longest inner one 20–24 μm, 1–2 minute setae on antennomere (Fig. 5D). Second antennomere wider (30–37 μm) than long (23–30 μm), with 8–9 setae inserted around antennal wall, interior setae (18–24 μm) longer than exterior ones (16–18 μm), one minute seta in some specimens (Fig. 5D). Chaetotaxy of third antennomere similar to that of the preceding ones (Fig. 5D). Setae on proximal antennomeres longer and on distal antennomeres shorter. Proximal antennomeres with only primary whorl of setae, on middle and subapical antennomeres with several minute setae in secondary whorl. Four types of sensory organs observed on antenna (Fig. 5D–F): *rso* on dorsal side of most antennomeres except first and apical antennomere; *so* only present on apical antennomere; *co* on dorsal side of antennomeres 3–6 to penultimate one, in some specimens both present on ventral and dorsal side on subterminal antennomere, seldom on apical antennomere, increasing in number to a maximum of six on subterminal antennomere; *bo* irregular, oblate, or curved, present on antennomere 5–9 to penultimate, increasing in number to a maximum of 17 on penultimate antennomere. Apical antennomere subspherical, as wide as long (length 25–30 μm, width 25–33 μm), five *so* and 16–20 setae on distal half (Fig. 5E, F). All antennomeres covered with short pubescence. Chaetotaxy and sensory organs of antennae of holotype are given in Table 5.

**Table 4.** Chaetotaxy of tergites of *Symphylella hylophila* sp. nov. (holotype in brackets).

**Tergite**	**Lateromarginal setae**	**Inserted seta**	**Central setae**	**Other setae**
1	4+4 or 3+4 (4+4)			
2	6–8 (6)	1–2 (1–2)	1–2 (2)	7–16 (9)
3	8–10 (10)	1–2 (1)	2–5 (3)	19–29 (23)
4	5–7 (5–6)	0–1 (0–1)	4–7 (4)	10–18 (11)
5	5–7 (6–7)	1–2 (1)	4–7 (4)	10–19 (12)
6	7–11 (9–10)	1–2 (1)	3–6 (4)	23–38 (28)
7	5–8 (7)	0–1 (1)	4–8 (5)	9–19 (13)
8	5–8 (7)	1–2 (1)	4–6 (6)	10–21 (10)
9	8–11 (8–9)	1–3 (1)	4–7 (5)	24–43 (27)
10	5–8 (6–7)	0–1 (0)	5–8 (5)	11–19 (14)
11	5–8 (5–6)	0–2 (1)	4–7 (5)	11–19 (11)
12	7–10 (9–10)	0–3 (1)	3–6 (5)	21–38 (27)
13	4–7 (5–6)	0–2 (0–1)	4–7 (5)	6–16 (10)
14				18–29 (20)
15	6–8 (6)	0–1 (1)	2–7 (2)	12–29 (15)
16				12–19 (14)
17				19–42 (19)

**Table 5.** Numbers of setae and sensory organs on antennae of *Symphylella rotundata* sp. nov. (holotype, excluding apical antennomere).

**Antennomere**	**Primary whorl setae**	**Secondary whorl setae**	**Rudimentary spined sensory organs**	**Cavity-shaped organs on dorsal side**	**Bladder-shaped organs**
1	6				
2	8		1		
3	8		1		
4	9		1		
5	10		1		
6	10			1	
7	10			1	1
8	11	2		1	1
9	11	5	1	1	1
10	11	5	1	1	1
11	11	5	1	1	1
12	11	5	1	1	3
13	11	5		1	5
14	11	5		1	7
15	11	5		1	12
16	11	3		3	15

***Trunk***. Length from base to tip of triangular processes the same as its basal width, except tergites 4, 7, 10, and 13 (shorter than basal width). Basal distance between processes of tergites distinctly longer than process length, except for tergites 2 and 3 (a little shorter than process length) (Table 6). All processes obviously with roundish ends (Fig. 4E, F). Anterolateral setae (26–52 μm) on tergites 2, 3, 4, 6, 7, 9, 10, and 12 distinctly longer than other *lms* of same tergite (Fig. 4C, D, G), while those on tergites 5, 8, and 11 (20–36 μm) shorter than longest third *lms* of same tergite (Fig. 4G). Each process with 1–3 *is*. All tergites pubescent (Fig. 4C, D, G).

**Table 6.** Measurements of tergites and processes of *Symphylella rotundata* sp. nov. (holotype in brackets, in μm).

**Tergite**	**Length**	**Width**	**Length of processes**	**Basal width of processes**	**Basal distance between processes**
1	25–37 (25)	115–140 (140)			
2	40–57 (57)	125–157 (157)	30–41 (41)	35–43 (43)	25–32 (30)
3	87–122 (122)	158–192 (192)	30–45 (45)	30–45 (45)	33–43 (43)
4	55–75 (75)	180–222 (222)	28–42 (40)	38–55 (50)	55–75 (75)
5	65–92 (92)	167–210 (210)	35–50 (50)	38–52 (52)	58–70 (70)
6	115–155 (155)	200–250 (250)	38–55 (55)	40–52 (52)	58–75 (75)
7	68–97 (97)	208–262 (262)	30–45 (45)	40–60 (60)	70–95 (90)
8	68–95 (95)	178–245 (245)	38–55 (55)	40–57 (57)	60–90 (82)
9	112–150 (112)	210–275 (275)	40–55 (55)	40–55 (55)	65–87 (87)
10	65–100 (100)	218–290 (290)	32–43 (37)	48–470 (55)	73–112 (112)
11	67–102 (102)	187–265 (265)	35–52 (52)	41–57 (57)	65–100 (100)
12	113–162 (162)	217–300 (300)	38–55 (55)	43–60 (60)	63–97 (90)
13	60–82 (82)	213–312 (312)	30–34 (34)	48–55 (55)	70–112 (112)
14	62–87 (87)	185–255 (255)			
15	102–120 (120)	203–275 (275)	25–40 (40)	40–57 (57)	50–75 (75)
16	60–75 (75)	170–205 (200)			
17	100–130 (120)	137–180 (180)			

***Tergites***. Tergite 1 reduced, with 4+3 setae in holotype, 4+4 or 4+5 in paratypes (Fig. 4C). Tergite 2 complete, with two triangular posterior processes, 6–8 *lms*, 1–2 *is*, 2–3 *cs*, *als* 0.8–1.4 of process length, processes 0.9–1.0 time as long as broad, basal distance between processes 0.7–1.0 as long as their length (Fig. 4C). Tergite 3 complete, broader and longer than preceding one, with ratios of 0.9–1.2, 0.9–1.1, and 0.8–1.6, respectively, 7–10 *lms*, 1–3 *is*, 2–4 *cs* (Fig. 4D). Tergite 4 broader than tergite 3, with ratios of 0.6–1.5, 0.7–0.8, and 1.4–2.4, respectively, 5–9 *lms*, 1–2 *is*, 3–5 *cs* (Fig. 4G). Chaetotaxy of tergites 5–7, 8–10, and 11–13 similar to tergites 2–4 (Fig. 4G). Pattern of alternating tergite lengths of two short tergites followed by one long tergite only disrupted at caudal end (Table 6). Tergites 14, 16, and 17 without processes, with 16–31, 10–20, and 22–42 setae, respectively. Measurements and chaetotaxy of tergites are given in Tables 6, 7, respectively.

***Legs***. First pair of legs reduced to two small hairy cupules, each with one long seta (11–12 μm) (Fig. 4H). Basal areas of legs 2–12 each with 3–10 setae. Leg 12 slightly shorter than head length, trochanter 1.0–1.7 times longer than wide (53–75 μm, 40–60 μm), with 7–8 subequal setae in total, longest seta 14–17 μm (Fig. 5I); femur almost as long as wide (33–45 μm, 33–48 μm), with 5–6 setae, longest dorsal seta (21–27 μm) 0.5–0.6 of greatest diameter of podomere in length, dorsally with cuticular thickenings in pattern of scales (Fig. 5J); tibia nearly 1.1–2.0 times longer than wide (35–60 μm, 25–33 μm), with 6–7 setae, longest dorsal one (20–24 μm) 0.7–0.9 of greatest diameter of tibia (Fig. 5J); tarsus sub-cylindrical, 3.5–4.8 times as long as wide (55–75 μm, 15–18 μm) with six dorsal setae: four straight and protruding, two depressed, longest seta (15–21 μm) slightly longer than greatest diameter of tarsus, two or three ventral setae distinctly shorter than dorsal ones. Claws slightly curved, anterior one distinctly longer than posterior one (Fig. 5J). All legs covered with dense pubescence except areas with cuticular thickenings.

***Coxal sacs*** present at bases of legs 3–9, fully developed, each with 4–6 setae on surface (Fig. 4I). Corresponding area of leg 2, 10, 11, and 12 replaced by 1–5 setae, respectively.

***Styli*** present at legs 3–12 base, short, subconical, length 8–9 μm, width 4–5 μm, basal part with dense straight hairs, distal quarter hairless, apex pointed (Figs 4I, 5G), on leg 12 slightly narrower (Fig. 5H).

***Sense calicles*** with smooth margin around pit. Sensory seta inserted in cup centre, extremely long (108–165 μm).

***Cerci*** about 0.5–0.6 time of head in length, 2.3–3.3 times as long as its greatest width (125–175 μm, 45–70 μm), densely covered with 68–107 setae. Two types of setae on surface: several long and erect setae located on outer, ventral, and dorsal side, other setae slightly curved and depressed (Fig. 5K, L). Longest outer long and erect seta (22–30 μm) not longer than half of greatest width of cerci, terminal area (20–25 μm) circled by 12–16 layers of curved ridges. Terminal seta (20–25 μm) same length with terminal area (Fig. 5K, L).

###### Etymology.

The species name *rotundata* is derived from the Latin adjective *rotundus*, meaning “round”, which refers to the roundish ends of the processes.

###### Distribution.

Inner Mongolia Autonomous Region and Shanxi Province (China).

###### Remarks.

*Symphylella rotundata* sp. nov. is similar to *S. yintiaolingensis* Jin & Bu, 2025 from Chongqing and *S. macrochaeta* Jin & Bu, 2023 from East China in the chaetotaxy of tergites and leg 12, as well as the shapes of the Tömösváry organ and the cercus. It differs from *S. yintiaolingensis* in the shape of the process (with a distinct roundish end in *S. rotundata* sp. nov. vs with a moderately swollen end in *S. yintiaolingensis*). It can be easily separated from *S. macrochaeta* by the chaetotaxy on the frons of the head (with 8 macrosetae arranged as 4/2/2 and in moderate length in *S. rotundata* sp. nov. vs with 10 extremely long macrosetae arranged as 4/4/2 in *S. macrochaeta*).

### DNA barcoding and genetic-distance analysis

DNA barcodes from eight specimens of the two new species were sequenced and submitted to GenBank (Table 1), each of them 658 base pairs in length. The pairwise genetic distance of 20 sequences of *Symphylella* species based on the K2P model are shown in Table 8. The genetic distance between *S. hylophila* sp. nov. and other congeners is 0.2177 on average (0.1685–0.2970). Although *S. rotundata* sp. nov. is very similar to *S. yintiaolingensis* and *S. macrochaeta* morphologically, the genetic distances among them are fairly high, with 0.1640 on average between *S. rotundata* sp. nov. and *S. yintiaolingensis*, and 0.1817 on average between *S. rotundata* sp. nov. and *S. macrochaeta* (Table 8). The genetic distance from *S. rotundata* sp. nov. to other congeners is 0.2021 on average (0.1550–0.3099), providing powerful support for our decision to describe it as a new species.

**Table 7.** Chaetotaxy of tergites of *Symphylella rotundata* sp. nov. (holotype in brackets).

**Tergite**	**Lateromarginal setae**	**Inserted seta**	**Central setae**	**Other setae**
1	3/4/5+4 (4+4)			
2	6–8 (7–8)	1–2 (1–2)	2–3 (2)	8–14 (12)
3	7–10 (8–9)	1–3 (2–3)	2–4 (2)	16–26 (16)
4	5–9 (6–7)	1–2 (2)	3–5 (4)	10–17 (16)
5	5–8 (7–8)	1–2 (2)	3–5 (3)	10–17 (14)
6	8–11 (8–9)	1–3 (2–3)	3–5 (4)	21–38 (30)
7	5–8 (6)	1–2 (2)	4–7 (5)	10–18 (17)
8	6–8 (7–8)	1–3 (3)	3–7 (4)	10–20 (16)
9	7–11 (9–10)	1–3 (2)	3–6 (4)	21–36 (33)
10	5–7 (6)	1–2 (1–2)	4–7 (5)	10–17 (17)
11	6–8 (7–8)	1–3 (1–2)	4–6 (4)	10–21 (35)
12	7–10 (9–10)	1–3 (2)	3–6 (4)	17–39 (35)
13	4–7 (6–7)	0–2 (0–2)	3–6 (6)	8–16 (16)
14				16–31 (31)
15	5–9 (6–7)	0–2 (2)	2–4 (4)	13–30 (30)
16				10–20 (18)
17				22–42 (38)

**Table 8.** Pairwise distances (K2P) between COI DNA sequences of *Symphylella* (interspecific distances in regular letters, intraspecific distances given in bold).

		**1**	**2**	**3**	**4**	**5**	**6**	**7**	**8**	**9**	**10**	**11**	**12**	**13**	**14**	**15**	**16**	**17**	**18**	**19**	**20**
1	PZ433329*Symphylella hylophila* sp. nov. LN-BX-2023014																				
2	PZ433330*Symphylella hylophila* sp. nov. LN-BX-2023015	**0.0015**																			
3	PZ433331*Symphylella rotundata* sp. nov. NM-2025018	0.1978	0.1978																		
4	PZ433332*Symphylella rotundata* sp. nov. NM-2025019	0.1917	0.1917	**0.0328**																	
5	PZ433333*Symphylella rotundata* sp. nov. NM-2025020	0.2019	0.2019	**0.0297**	**0.0281**																
6	PZ433334*Symphylella rotundata* sp. nov. NM-2025021	0.1938	0.1938	**0.0344**	**0.0046**	**0.0329**															
7	PZ433335*Symphylella rotundata* sp. nov. NM-2025022	0.1917	0.1917	**0.0378**	**0.0329**	**0.0170**	**0.0345**														
8	PZ433336*Symphylella rotundata* sp. nov. SX-DT-2023004	0.2019	0.2019	**0.0361**	**0.0265**	**0.0281**	**0.0281**	**0.0297**													
9	PX169704*Symphylella communa* JS-WX-2021010	0.2044	0.2064	0.2059	0.1958	0.1897	0.1978	0.1978	0.2019												
10	PX169697*Symphylella flabella* CQ-YTL-2022007	0.1685	0.1685	0.1629	0.1611	0.1553	0.1670	0.1631	0.1592	0.1890											
11	PX169702*Symphylella macrochaeta* SH-JZGY-2021009	0.2297	0.2297	0.1857	0.1917	0.1819	0.1897	0.1740	0.1859	0.2149	0.2190										
12	PX169703*Symphylella macrochaeta* ZJ-ZS-2020011	0.2215	0.2215	0.1803	0.1861	0.1763	0.1841	0.1646	0.1803	0.2054	0.2088	**0.0438**									
13	PX169698*Symphylella micropora* CQ-JYS-2022001	0.2181	0.2202	0.1689	0.1689	0.1788	0.1709	0.1768	0.1650	0.2195	0.1689	0.2309	0.2230								
14	PX169700*Symphylella micropora* CQ-YTL-2022009	0.2202	0.2223	0.1709	0.1709	0.1809	0.1728	0.1788	0.1669	0.2195	0.1709	0.2309	0.2230	**0.0030**							
15	PX169705*Symphylella minuta* JS-WX-2021008	0.2756	0.2780	0.2232	0.2210	0.2276	0.2188	0.2232	0.2232	0.2381	0.2457	0.2573	0.2389	0.2432	0.2432						
16	PX169693*Symphylella obtusa* CQ-JYS-2021002	0.2970	0.2945	0.2852	0.3024	0.2999	0.2999	0.3099	0.2901	0.2799	0.2735	0.2961	0.2920	0.3078	0.3078	0.2862					
17	PX169694*Symphylella obtusa* CQ-JYS-2021012	0.2899	0.2875	0.2736	0.2903	0.2928	0.2879	0.2977	0.2783	0.2844	0.2684	0.2983	0.2941	0.2955	0.2955	0.2866	**0.0154**				
18	PX169695*Symphylella yintiaolingensis* YTL-2022004	0.1859	0.1859	0.1703	0.1550	0.1666	0.1569	0.1685	0.1666	0.1909	0.1453	0.2000	0.1942	0.1750	0.1750	0.2129	0.2991	0.2919			
19	NC011572*Symphylella* sp. YG-2006	0.2095	0.2095	0.1949	0.2009	0.1949	0.1988	0.1847	0.1970	0.2064	0.1766	0.1062	0.0822	0.2002	0.2002	0.2369	0.2793	0.2772	0.1943		
20	OQ165321*Scutigerella sinensis* JYL-DJS2017011	0.3127	0.3127	0.2918	0.3031	0.3078	0.3008	0.2985	0.3031	0.3107	0.2776	0.3195	0.2987	0.3416	0.3416	0.3649	0.3401	0.3322	0.3113	0.2947	

Intraspecific distances are known only for a few species in *Symphylella* and are 0.0216 on average (0.0015–0.0438) in our dataset. The intraspecific distance of *S. hylophila* sp. nov. between two individuals is only 0.0015, but this species is known from a single population. The intraspecific distance between individuals from five known populations of *S. rotundata* sp. nov. is 0.0289 on average (0.0046–0.0378). The intraspecific distances of the remaining species of the genus are likewise low, 0.0438 in *S. macrochaeta* between the populations from neighbouring regions, 0.0030 in *S. micropora* between the populations from the same province, and 0.0154 in *S. obtusa* between the individuals from the same population (Table 8).

The interspecific distance between 10 species of the genus *Symphylella* is 0.2165 on average (range: 0.0822–0.3099). The smallest value, 0.0822, is the distance between *Symphylella* sp. from Jiangsu and *S. macrochaeta* from Shanghai, which is much lower than the average value, indicating a close relationship between them. *Symphylella* species have the highest genetic distances compared with *Scutigerella sinensis* from the family Scutigerellidae, with a value of 0.3139 on average (range: 0.2776–0.3649) (Table 8).

**Figure 6.**
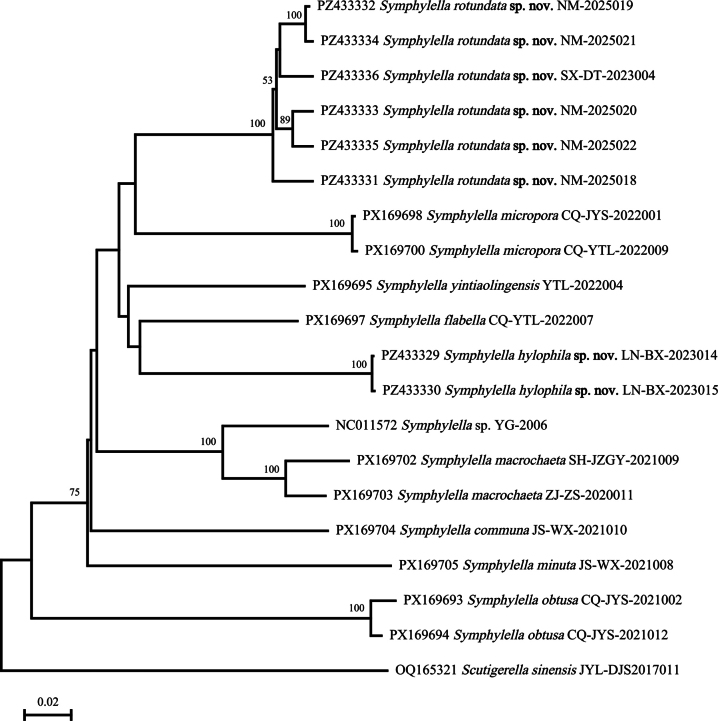
Neighbour-joining tree (K2P-distance, Bootstrap 1000 replicates) of *Symphylella* inferred from COI gene sequences. Numbers on the nodes show the bootstrap values (> 50%).

Based on COI gene sequences, a neighbour-joining tree was constructed for 10 species of *Symphylella* (Fig. 6), and each species separated in a monophyletic cluster (Fig. 6). All populations for *S. rotundata* sp. nov. cluster together, and two individuals of *S. hylophila* sp. nov. formed a unique clade, which further supports our taxonomic decision.

## Discussion

Symphyla is poorly known from North China, with only two species previously recorded: *Symphylella longispina* Jin & Bu, 2023 from Xinjiang Uygur Autonomous Region and *Scutigerella sinensis* Jin & Bu, 2023 from Beijing. The two new species described in the present study further enrich the knowledge on symphylan diversity in North China. *Symphylella hylophila* sp. nov. is only known from the broad-leaf forest of Liaoning Province, Northeast China, while *Symphylella rotundata* sp. nov. was found in arid sandy soil and litter across central and eastern Inner Mongolia Autonomous Region and Shanxi Province, which indicates that this species is probably adapted to arid habitats in North China.

DNA barcoding results confirm the validation of the two new *Symphylella* species at the molecular level. The interspecific genetic distances among all recognized species of *Symphylella* are distinctly higher than intraspecific distances in this genus, with a lowest interspecific genetic distance of 0.0822 and a highest intraspecific genetic distance of 0.0438. These two values may tentatively deemed as thresholds for species delimitation within the genus. At the very least, individuals with a genetic distance below 0.0438 can be identified as conspecific, while taxa with a genetic distance above 0.0822 belong to different species. Admittedly, those two thresholds are preliminary results based on limited available data, and more molecular data for more species are required to determine the precise genetic distance thresholds.

Notably, although *S. rotundata* sp. nov. exhibits highly similar morphological characteristics to *S. yintiaolingensis* and *S. macrochaeta*, the considerable interspecific genetic distances clearly distinguish *S. rotundata* sp. nov. from its morphologically similar relatives. Therefore, molecular data can effectively compensate for the limitations of traditional morphological identification, further validating the taxonomic validity of the new species, and enrich the data basis for future species identification and phylogenetic study of Symphyla.

The genus *Symphylella* has a worldwide distribution, with a high number of described species from different continents. Some species are morphologically very similar to each other and can only be separated by several extremely subtle characters, such as the shape of tergal processes and of the stylus, and the chaetotaxy of the cercus. The present study proves that DNA barcoding offers powerful evidence for species delimitation among *Symphylella* species, supplementing morphology. We suggest that molecular data once provided for a higher number of symphylan species, will greatly improve our understanding of this understudied group of soil arthropods.

## Supplementary Material

XML Treatment for Symphylella

XML Treatment for Symphylella hylophila

XML Treatment for Symphylella rotundata

## Additional information

### Artificial Intelligence (AI) use

The authors accept full responsibility for the content of the manuscript, including the disclosure of any use of AI.

No AI tools were used in the preparation of this manuscript.

### Author ORCIDs

Y.-L. Jin https://orcid.org/0009-0003-5773-2165

Y. Bu https://orcid.org/0000-0002-7177-9686

### Data availability

All of the data that support the findings of this study are available in the main text.
